# The Association Between Atherogenic Index of Plasma and Type 2 Diabetes Mellitus in a Multi‐Ethnic Population of Premature Coronary Artery Disease Patients: Insights From IPAD Study

**DOI:** 10.1002/edm2.70280

**Published:** 2026-08-12

**Authors:** Reza Amani‐Beni, Ghazal Ghasempour Dabaghi, Noushin Mohammadifard, Ehsan Zarepur, Bahar Darouei, Mehrdad Rabiee Rad, Fahimeh Haghighatdoost, Fatemeh Nouri, Nizal Sarrafzadegan

**Affiliations:** ^1^ Isfahan Cardiovascular Research Center, Cardiovascular Research Institute Isfahan University of Medical Sciences Isfahan Iran; ^2^ Interventional Cardiology Research Center, Cardiovascular Research Institute Isfahan University of Medical Sciences Isfahan Iran; ^3^ Heart Failure Research Center, Cardiovascular Research Institute Isfahan University of Medical Sciences Isfahan Iran; ^4^ Iranian Network of Cardiovascular Research (INCVR) Tehran Iran

**Keywords:** atherogenic index of plasma, coronary artery disease, ethnicity, insulin resistance, type 2 diabetes mellitus

## Abstract

**Background:**

The atherogenic index of plasma (AIP) is a lipid‐based indicator of cardiometabolic risk. We examined whether AIP is associated with prevalent type 2 diabetes mellitus (T2DM) and fasting blood sugar (FBS) in patients with premature coronary artery disease (PCAD) and explored whether this association differed across Iranian ethnic groups.

**Methods:**

We analysed 2143 patients with angiographically confirmed PCAD from the Iran Premature Coronary Artery Disease (IPAD) study, of whom 1945 had valid AIP data. CAD was defined as ≥ 50% stenosis in the left main artery or ≥ 75% stenosis in another major coronary vessel. AIP was calculated as log_10_ (triglycerides/high‐density lipoprotein cholesterol). Multivariable logistic regression was used to assess the association between AIP and prevalent T2DM, and generalized linear models were used to assess the association between AIP and FBS.

**Results:**

In the fully adjusted model, including lipid‐lowering medication use, participants in the highest AIP quartile had higher odds of prevalent T2DM than those in the lowest quartile (OR: 1.57; 95% CI: 1.14, 2.16; *p* = 0.005; P for trend = 0.002). Each 1‐unit increase in AIP was associated with higher odds of prevalent T2DM (OR: 1.99; 95% CI: 1.26, 3.14; *p* = 0.003). Higher AIP was also associated with higher FBS; compared with the lowest quartile, the highest quartile had a 15.23 mg/dL higher FBS level in the fully adjusted model (95% CI: 7.61, 22.84; *p* < 0.001). In exploratory ethnicity‐stratified analyses, the association was most evident in the Fars subgroup, while estimates in several smaller ethnic groups were imprecise.

**Conclusions:**

Higher AIP was associated with prevalent T2DM and higher FBS in patients with PCAD. Exploratory analyses suggested possible ethnicity‐related heterogeneity, but these subgroup findings should be interpreted cautiously. Prospective studies are needed to determine whether AIP has predictive or clinical risk‐stratification value in this population.

## Graphical abstract

In patients with premature coronary artery disease, higher atherogenic index of plasma was associated with prevalent type 2 diabetes and higher fasting blood sugar. Exploratory analyses suggested possible ethnicity‐related heterogeneity, while ROC analysis showed limited discrimination, supporting cautious interpretation rather than clinical screening use.

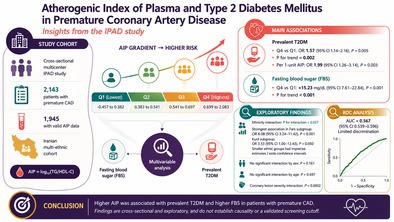

## 1 Introduction

Type 2 diabetes mellitus (T2DM) is a major global health problem because it drives both small‐ and large‐vessel complications, and its prevalence is projected to reach hundreds of millions of people worldwide in the coming decades [1, 2]. T2DM is an independent risk factor for heart disease since this condition leads to 2–4 times higher chance of cardiovascular diseases (CVDs), a major macrovascular consequence that includes coronary artery disease (CAD) and myocardial infarction (MI) [3]. T2DM and CAD are closely linked together in a way that T2DM can be described as a coronary risk equivalent, and it has been reported that 34.8% and 17.7% of patients with T2DM have CVD and CAD, respectively [4, 5]. In clinical cohorts, diabetes is present in a large proportion of patients with CAD, and when these two conditions coexist, cardiovascular events occur earlier and outcomes are worse, particularly in younger adults [6, 7]. These alarming statistics emphasize the urgent need for cardiovascular risk decline in these patients, especially in those with established CAD [2].

Premature CAD (PCAD) generally refers to clinically significant coronary disease occurring at younger‐than‐expected ages, although age thresholds vary across studies and cohorts [8]. PCAD can cause a significant economic burden on healthcare as it reduces the potential years of life, and accompanying T2DM with PCAD can multiply this burden by accelerating the onset of cardiovascular events [9]. In particular, there is clinical value in identifying inexpensive markers associated with prevalent dysglycemia and cardiometabolic burden in the PCAD setting.

Among traditional risk factors, lipid‐derived indices may be associated with insulin resistance and dysglycemia, as they are significantly and independently associated with insulin resistance (IR) [10, 11]. Dobiášová and Frohlich were the first to establish the lipid profile known as the atherogenic index of plasma (AIP) [12]. Since AIP fully illustrates the balance between various lipoproteins and plasma atherogenicity, it has been the subject of numerous research as a prognostic marker [13, 14, 15]. Higher AIP levels have been found to substantially raise the risk of major adverse cardiovascular events (MACE) and CVD death in individuals with CAD, according to a recent meta‐analysis [13]. Furthermore, because high AIP considerably raises the risk of arterial stiffness, atherosclerosis, and hypertension, it has been demonstrated to be prognostic in T2DM patients [16].

An important topic of research in cardiovascular and metabolic health is the relationship between AIP and prevalent T2DM in patients with PCAD. This question is clinically relevant, because diabetes alters management decisions and prognosis in young CAD patients. Hence, in this multi‐ethnic study, we aimed to evaluate the association between AIP values and prevalent T2DM among patients with PCAD. In addition, we evaluated the association of AIP with fasting blood sugar (FBS) in these patients and performed a subgroup analysis based on ethnicity.

## 2 Materials and Methods

### 2.1 Study Design and Population

This cross‐sectional study draws on the Iran Premature Coronary Artery Disease (IPAD) study, a multicenter case–control study that enrolled patients from 15 Iranian cities representing several major ethnic groups. The IPAD sampling strategy, recruitment process, and core protocol have been published in detail elsewhere [17]. Briefly, adults referred for diagnostic coronary angiography were screened. Eligible women were ≤ 70 years old and eligible men were ≤ 60 years old, and all participants self‐identified with one of the predefined Iranian ethnicities. Individuals were classified as CAD cases if angiography showed ≥ 75% stenosis in at least one major epicardial vessel or ≥ 50% stenosis of the left main coronary artery. Participants with angiographically normal coronary arteries served as controls. We excluded anyone with a prior history of clinically documented CAD, including prior percutaneous coronary intervention or coronary artery bypass grafting. For the current analysis, we included 2143 participants who met criteria for PCAD, of whom 1945 had valid AIP values and were included in analyses across AIP quartiles. All participants signed written informed consent, and the study was approved by the Ethics Committee of Isfahan University of Medical Sciences (IR.MUI.REC.1396.2.055).

### 2.2 Data Collection

Enrollment was coordinated through catheterization laboratories that predominantly serve specific ethnic communities to ensure representation of all targeted ethnic groups. Trained staff used the standardized IPAD case report form [17] to obtain sociodemographic information (age, sex, self‐reported ethnicity, education, marital status) and lifestyle factors (current smoking status and alcohol intake). Physical activity was assessed using the validated Iranian version of the International Physical Activity Questionnaire (IPAQ) [18].

Anthropometrics were obtained by trained personnel using a unified protocol. Height was measured to the nearest 0.1 cm with a wall‐mounted stadiometer, and weight was recorded to the nearest 0.1 kg with participants wearing light clothing and no shoes. Body mass index (BMI) was calculated as weight (kg) divided by height (m^2^). Waist circumference (WC) was measured midway between the lowest rib and the iliac crest and recorded to 0.1 cm accuracy [19].

For the present analysis, lipid‐lowering medication use was defined as current use of any statin, fibrate, or ezetimibe. Glucose‐lowering medication use was defined as current use of oral hypoglycemic agents or insulin and was used as part of the T2DM definition.

After an overnight fast of approximately 12 h, venous blood (10 mL) was drawn. Samples were centrifuged (3000 rpm, 10 min) and stored at −80°C until analysis. Each participating center measured FBS, total cholesterol (TC), and triglycerides (TG) using enzymatic assays and commercial kits from the same manufacturer (Pars Azmoon Co., Tehran, Iran). High‐density lipoprotein cholesterol (HDL‐C) was quantified after precipitation of non‐HDL lipoproteins with dextran sulfate–magnesium chloride, using a standard method [20]. All biochemical measurements were performed according to standard laboratory quality‐control procedures, including routine monitoring of intra‐assay and inter‐assay variability. We defined hypercholesterolemia according to Adult Treatment Panel III criteria (TC ≥ 240 mg/dL) [21]. T2DM was defined using American Diabetes Association diagnostic thresholds: FBS ≥ 126 mg/dL, 2‐h glucose > 200 mg/dL, or current use of glucose‐lowering medication [22]. AIP was computed as log_10_ (TG/HDL‐C) [12].

### 2.3 Statistical Analysis

Continuous variables are summarized as mean ± standard deviation (SD), and categorical variables are presented as counts and percentages. We compared baseline characteristics across AIP quartiles using a one‐way analysis of variance (ANOVA) for continuous variables and the chi‐square test for categorical variables.

To study the relationship between AIP and T2DM, we ran logistic regression models that treated the lowest AIP quartile as the reference category. We also modelled AIP as a continuous predictor. Associations between AIP quartiles and FBS were assessed using generalized linear models (GLM). Covariate adjustment was performed in four stages. Model 1 was unadjusted. Model 2 controlled for age and sex. Model 3 additionally included education, smoking status, alcohol intake, physical activity, and marital status. Model 4 further adjusted for central and general adiposity, including waist circumference and BMI, hypertension, hypercholesterolemia, and lipid‐lowering medication use. Glucose‐lowering medication use was not added as a separate covariate in the T2DM models because it was part of the outcome definition. Multicollinearity among covariates in the fully adjusted model was assessed using variance inflation factors (VIFs).

Because several ethnic subgroups had relatively small sample sizes and sparse T2DM events, ethnicity‐stratified analyses were considered exploratory. To reduce sparse‐data bias, AIP was modelled as a continuous variable in ethnicity‐stratified analyses. For each ethnicity, we fitted a crude model and an age‐ and sex‐adjusted model. We also tested the overall AIP‐by‐ethnicity interaction in the full sample. Participants with other or unknown ethnicity were excluded from ethnicity‐stratified regression because of very small sample size.

Additional exploratory subgroup analyses were performed by sex, age group (< 55 vs. ≥ 55 years), and coronary lesion severity. In these analyses, AIP was modelled as a continuous variable, and interaction terms were used to assess whether the association between AIP and prevalent T2DM differed across subgroups. Coronary lesion severity was categorized as 1‐vessel, 2‐vessel, or 3‐vessel disease. An exploratory ROC analysis was also performed to assess the discriminatory performance of AIP alone for prevalent T2DM. Because this ROC analysis was exploratory and no external validation was available, no clinical cutoff was derived.

We report odds ratios (ORs) and 95% confidence intervals (CIs). Analyses were performed using Stata version 18.0 (StataCorp LLC, College Station, TX, USA). A two‐sided *p* < 0.05 was considered statistically significant.

## 3 Results

Among 2143 patients with PCAD, 1945 had valid AIP values and were included in the analysis across AIP quartiles. Of these, 65.6% were men, and the mean age was approximately 55 years (SD 7). Table 1 summarizes baseline characteristics according to AIP quartiles. The AIP quartile ranges were −0.457 to 0.382 for Q1, 0.383 to 0.541 for Q2, 0.541 to 0.697 for Q3, and 0.699 to 2.083 for Q4. Participants in higher AIP quartiles tended to be younger and more often male, and had higher BMI and waist circumference. They were also more likely to have T2DM and hypercholesterolemia. The distribution of ethnicity and lipid‐lowering medication use differed significantly across AIP quartiles. In contrast, marital status, smoking, alcohol intake, physical activity, education, and hypertension did not significantly differ by AIP category.

**TABLE 1:** General and demographic data in participants with premature coronary artery disease based on quartiles of AIP.

Characteristics	AIP quartiles				*p*
	Q1 (*n* = 487)	Q2 (*n* = 487)	Q3 (*n* = 485)	Q4 (*n* = 486)	
AIP range	−0.457 to 0.382	0.383 to 0.541	0.541 to 0.697	0.699 to 2.083	
Male, *n* (%)	296 (60.8)	318 (65.3)	325 (67.0)	337 (69.3)	**0.037**
Age, years (mean ± SD)	56 ± 7	55 ± 7	55 ± 7	54 ± 7	**0.008**
Married, *n* (%)	434 (89.1)	442 (90.8)	437 (90.1)	444 (91.4)	0.673
Current smoking, *n* (%)	113 (23.2)	109 (22.4)	114 (23.6)	125 (25.7)	0.651
Alcohol drinking, *n* (%)	21 (4.3)	12 (2.5)	27 (5.6)	27 (5.6)	0.061
Ethnicity					**0.014**
Fars	272 (55.9)	291 (59.8)	260 (53.6)	233 (47.9)	
Azari	46 (9.5)	38 (7.8)	41 (8.5)	48 (9.9)	
Kurd	54 (11.1)	36 (7.4)	50 (10.3)	51 (10.5)	
Lor	27 (5.5)	32 (6.6)	35 (7.2)	40 (8.2)	
Bakhtiari	24 (4.9)	24 (4.9)	45 (9.3)	40 (8.2)	
Qashqaei	20 (4.1)	16 (3.3)	19 (3.9)	20 (4.1)	
Gilak	39 (8.0)	45 (9.2)	31 (6.4)	54 (11.1)	
Other/unknown	5 (1.0)	5 (1.0)	4 (0.8)	0 (0.0)	
Education *n* (%)					0.525
Less than high school	261 (53.7)	247 (50.7)	235 (48.5)	240 (49.4)	
High school or college	175 (36.0)	191 (39.2)	186 (38.4)	187 (38.5)	
University	50 (10.3)	49 (10.1)	64 (13.2)	59 (12.1)	
Physical activity, MET‐hour/week	29.51 ± 53.41	35.60 ± 70.64	32.28 ± 68.36	32.38 ± 51.02	0.495
BMI, kg/m^2^	27.14 ± 4.88	27.92 ± 5.34	28.52 ± 4.37	28.78 ± 4.92	**< 0.001**
Waist circumference, cm	102.48 ± 11.28	103.43 ± 11.02	104.76 ± 9.29	104.26 ± 11.46	**0.006**
Hypertension, *n* (%)	185 (40.7)	194 (43.2)	207 (46.1)	214 (48.0)	0.128
Diabetes mellitus, *n* (%)	119 (28.1)	143 (34.9)	176 (41.9)	176 (41.8)	**< 0.001**
Hypercholesterolemia, *n* (%)	130 (28.5)	149 (33.3)	179 (39.7)	201 (45.1)	**< 0.001**
Lipid‐lowering medication use, *n* (%)	392 (81.7)	395 (82.3)	362 (76.1)	368 (77.8)	**0.046**

*Note:* Bold values indicate statistical significance (*p* < 0.05).

Abbreviations: AIP, atherogenic index of plasma; BMI, body mass index; Q, quartile; SD, standard deviation; WC, waist circumference.

Table 2 shows the association between AIP quartiles and FBS among patients with PCAD. In the crude model, patients in the highest AIP quartile had a 21.13 mg/dL higher FBS level compared with those in the lowest quartile (*β* = 21.13; 95% CI: 13.82, 28.44; *p* < 0.001; *p* for trend < 0.001). After full adjustment, including lipid‐lowering medication use, the association remained significant. Compared with the first quartile, the fourth AIP quartile was associated with a 15.23 mg/dL higher FBS level (*β* = 15.23; 95% CI: 7.61, 22.84; *p* < 0.001; *p* for trend < 0.001).

**TABLE 2:** Association of fasting blood sugar with AIP among premature coronary artery disease patients.

AIP	Q1	Q2		Q3		Q4		*p* for linear trend
	*β* (95% CI)	*β* (95% CI)	*p*	*β* (95% CI)	*p*	*β* (95% CI)	*p*	
Model 1	Reference	6.31 (−0.99, 13.61)	0.090	11.83 (4.52, 19.15)	**0.002**	21.13 (13.82, 28.44)	**< 0.001**	**< 0.001**
Model 2	Reference	6.89 (−0.35, 14.13)	0.062	12.76 (5.49, 20.02)	**0.001**	22.74 (15.47, 30.02)	**< 0.001**	**< 0.001**
Model 3	Reference	6.77 (−0.49, 14.02)	0.068	13.02 (5.74, 20.30)	**< 0.001**	22.94 (15.66, 30.22)	**< 0.001**	**< 0.001**
Model 4	Reference	6.47 (−0.95, 13.88)	0.088	10.58 (3.13, 18.03)	**0.005**	15.23 (7.61, 22.84)	**< 0.001**	**< 0.001**

*Note:* Model 1: Crude model. Model 2: Adjusted for age and sex. Model 3: Additionally, adjusted for education, smoking (never/ex‐smoker/current smoker), alcohol consumption, physical activity (MET‐hour/week), and marital status. Model 4: Additionally, adjusted for waist circumference, body mass index (Kg/m^2^), hypertension (yes/no), hypercholesterolemia (yes/no), and lipid‐lowering medication use. Bold values indicate statistical significance (*p* < 0.05).

Abbreviations: AIP, atherogenic index of plasma; FBS, fasting blood sugar; *p*, *p* value; Q, quartile.

Table 3 presents the association between AIP and prevalent T2DM among patients with PCAD. In the crude model, participants in the highest AIP quartile had higher odds of T2DM than those in the lowest quartile (OR = 1.84; 95% CI: 1.38, 2.45; *p* < 0.001; *p* for trend < 0.001). After full adjustment, including lipid‐lowering medication use, the association remained significant. Compared with Q1, the adjusted ORs were 1.34 (95% CI: 0.98, 1.84) for Q2, 1.67 (95% CI: 1.22, 2.28) for Q3, and 1.57 (95% CI: 1.14, 2.16) for Q4 (*p* for trend = 0.002). When AIP was modelled as a continuous variable, each 1‐unit increase in AIP was associated with higher odds of prevalent T2DM in the fully adjusted model (OR = 1.99; 95% CI: 1.26, 3.14; *p* = 0.003). VIF analysis did not indicate problematic multicollinearity among covariates in the fully adjusted model; all VIF values were below 2.

**TABLE 3:** Odds ratio (95% confidence interval) of prevalent diabetes mellitus according to quartiles of AIP.

AIP	Per 1‐unit increase		Q1	Q2		Q3		Q4		*p* for linear trend
	OR (95% CI)	*p*	OR (95% CI)	OR (95% CI)	*p*	OR (95% CI)	*p*	OR (95% CI)	*p*	
Model 1	2.47 (1.63, 3.73)	**< 0.001**	Reference	1.37 (1.02, 1.84)	**0.036**	1.84 (1.38, 2.46)	**< 0.001**	1.84 (1.38, 2.45)	**< 0.001**	**< 0.001**
Model 2	3.00 (1.96, 4.60)	**< 0.001**	Reference	1.44 (1.07, 1.95)	**0.017**	2.03 (1.51, 2.73)	**< 0.001**	2.08 (1.54, 2.79)	**< 0.001**	**< 0.001**
Model 3	3.12 (2.03, 4.79)	**< 0.001**	Reference	1.45 (1.07, 1.96)	**0.017**	2.03 (1.51, 2.74)	**< 0.001**	2.11 (1.57, 2.84)	**< 0.001**	**< 0.001**
Model 4	1.99 (1.26, 3.14)	**0.003**	Reference	1.34 (0.98, 1.84)	0.070	1.67 (1.22, 2.28)	**0.001**	1.57 (1.14, 2.16)	**0.005**	**0.002**

*Note:* Model 1: Crude model. Model 2: Adjusted for age and sex. Model 3: Additionally, adjusted for education, smoking (never/ex‐smoker/current smoker), alcohol consumption, physical activity (MET‐hour/week), and marital status. Model 4: Additionally, adjusted for waist circumference, body mass index (Kg/m^2^), hypertension (yes/no), hypercholesterolemia (yes/no), and lipid‐lowering medication use. Bold values indicate statistical significance (*p* < 0.05).

Abbreviations: AIP, atherogenic index of plasma; OR, odds ratio; *p*, *p* value; Q, quartile.

Table 4 presents the exploratory ethnicity‐stratified association between continuous AIP and prevalent T2DM. In the age‐ and sex‐adjusted model, higher AIP was significantly associated with higher odds of T2DM in the Fars subgroup (OR = 6.08; 95% CI: 3.24, 11.42; *p* < 0.001). A borderline association was observed in the Kurd subgroup (OR = 3.53; 95% CI: 1.00, 12.43; *p* = 0.050). Estimates in the other ethnic groups were not statistically significant and had wide confidence intervals. The overall age‐ and sex‐adjusted interaction between AIP and ethnicity was statistically significant (*p* for interaction = 0.027), suggesting possible ethnicity‐related heterogeneity. However, because several ethnic subgroups had small sample sizes and sparse events, these findings should be interpreted as exploratory and hypothesis‐generating.

**TABLE 4:** Ethnicity‐stratified association between AIP and prevalent T2DM.

Ethnicity	*n*/T2DM cases	Model 1 OR per 1‐unit AIP (95% CI)	*p*	Model 2 OR per 1‐unit AIP (95% CI)	*p*
Fars	923/343	4.62 (2.52, 8.47)	**< 0.001**	6.08 (3.24, 11.42)	**< 0.001**
Azari	110/50	1.20 (0.28, 5.11)	0.802	1.29 (0.30, 5.61)	0.735
Kurd	177/52	3.30 (0.97, 11.18)	0.056	3.53 (1.00, 12.43)	**0.050**
Lor	112/37	1.93 (0.44, 8.47)	0.383	2.23 (0.45, 11.02)	0.324
Bakhtiari	111/30	5.27 (0.94, 29.47)	0.058	5.54 (0.86, 35.77)	0.072
Qashqaei	68/19	1.69 (0.21, 13.58)	0.623	2.34 (0.26, 21.08)	0.447
Gilak	159/78	0.43 (0.12, 1.48)	0.180	0.44 (0.12, 1.62)	0.214

*Note:* AIP was modeled as a continuous variable. Model 1 was crude. Model 2 was adjusted for age and sex. Other/unknown ethnicity was excluded from ethnicity‐stratified analyses because of very small sample size. These analyses were considered exploratory because several ethnic subgroups had limited sample sizes and sparse T2DM events, particularly the Qashqaei subgroup. Bold values indicate statistical significance (*p* < 0.05).

Abbreviations: AIP, atherogenic index of plasma; CI, confidence interval; OR, odds ratio; T2DM, type 2 diabetes mellitus.

Additional exploratory subgroup analyses by sex, age group, and coronary lesion severity are presented in Table S1. No statistically significant interaction was observed by sex (*p* for interaction = 0.161) or age group (*p* for interaction = 0.697). The AIP‐by‐coronary lesion severity interaction was statistically significant (*p* for interaction = 0.0002). In exploratory ROC analysis, AIP alone showed limited discrimination for prevalent T2DM (AUC = 0.567; 95% CI: 0.539, 0.596; Figure S1).

## 4 Discussion

In this multicenter cross‐sectional sample of patients with PCAD, higher AIP was associated with prevalent T2DM and higher FBS. These associations persisted after adjustment for demographic, lifestyle, cardiometabolic covariates, and lipid‐lowering medication use, and were also observed when AIP was modelled as a continuous measure. Because of the cross‐sectional design, these findings should be interpreted as associations with prevalent dysglycemia rather than evidence of causality, prediction, or screening utility.

These results are clinically important because PCAD occurs at relatively young ages and therefore carries a disproportionate long‐term burden in terms of recurrent events, disability, and loss of productive life‐years [23]. Coexistence of type 2 diabetes in this setting further worsens prognosis, since diabetes accelerates atherosclerotic plaque formation, increases multivessel and obstructive disease, and is associated with higher cardiovascular event rates and mortality compared with nondiabetic patients of similar age [24, 25]. This makes early recognition of unrecognized or poorly controlled metabolic disease a priority in secondary prevention for younger CAD patients [25, 26]. In this context, our findings suggest that AIP is associated with the burden of dysglycemia among patients with PCAD, although prospective studies are needed before it can be used for prediction or clinical decision‐making [27].

Our findings are consistent with previous longitudinal studies that reported associations between higher AIP and future T2DM risk in non‐PCAD populations. However, unlike those studies, our analysis was cross‐sectional and therefore cannot determine whether higher AIP precedes the development of T2DM [28, 29, 30]. However, a limited number of studies have evaluated this index in PCAD patients. Large cohort data have shown that AIP is related to incident diabetes in middle‐aged adults of both sexes, especially in the 40–64 year range [28]. In a separate study of predominantly middle‐aged and older adults (84.8% were between 45 and 69 years old), higher AIP values were linked to a 34% higher likelihood of developing T2DM [29]. Likewise, findings from a retrospective cohort showed that elevated AIP was associated with progression from prediabetes to overt T2DM, while higher AIP was inversely related to regression from prediabetes back to normal glycemia [30].

We also found that FBS increased across AIP quartiles, indicating that poorer glycemic status is linked to a more atherogenic lipid profile in patients with PCAD. This is consistent with prior metabolic evidence. Li et al. reported that in 2523 individuals with diabetes, higher AIP corresponded to greater IR and was directly related to FBS, 2‐h post‐load glucose, and HbA1c, supporting the idea that AIP reflects global glycemic dysregulation rather than lipids alone [31]. In another cohort of 4252 adults with established T2DM, higher AIP tracked with higher HbA1c and higher FBS, and this relationship was even stronger than that observed for non–HDL cholesterol, suggesting that AIP may outperform traditional lipid measures as an indicator of poor glycemic control in diabetes [32].

Elevated TG, decreased HDL‐C, and an excess of small, dense LDL particles are the typical lipid patterns associated with T2DM, also known as diabetic dyslipidemia [33]. This profile is metabolically unfavourable. High TG levels are closely linked to IR, which impairs cellular glucose handling and oxidation, and may downregulate both the activity and density of insulin receptors in adipose tissue [33]. Low HDL‐C has also been tied to impaired β‐cell function, with reductions in both insulin secretion and insulin sensitivity [33]. IR can worsen this lipid pattern, and the lipid abnormalities in turn can further aggravate IR, creating a self‐reinforcing loop between dyslipidemia and disordered glucose metabolism that is known by the term “vicious cycle” in diabetes [28, 34]. Thus, a meta‐analysis of 15 studies comparing the predictive power of these lipid profiles for the risk of developing T2DM, found that while other lipid parameters like TG, HDL‐C, LDL‐C, and TC can significantly reflect the risk of developing T2DM, AIP is more strongly and closely linked to this risk [35]. It can be suggested that because AIP mathematically integrates triglycerides and HDL‐C into a single log‐transformed ratio, it captures this atherogenic, insulin‐resistant profile in a compact way.

On the other hand, AIP is not only linked to glucose dysregulation, it's consistently associated with other cardiometabolic risk features like central adiposity (waist circumference), IR, high TG, and low HDL‐C, the core components of metabolic syndrome [27]. Although proposed AIP cut ranges have been discussed for cardiovascular risk, these cutoffs have not been validated for identifying T2DM among patients with PCAD, and our cross‐sectional data cannot establish clinical screening utility [27]. Elevated AIP has also been tied to arterial stiffness and adverse vascular remodelling, which are early manifestations of endothelial injury and subclinical atherosclerosis and are known predictors of future cardiovascular events [36, 37]. In clinical populations, higher AIP has been independently associated with major adverse cardiovascular events (including myocardial infarction and stroke) and with angiographically defined obstructive CAD, even after accounting for traditional risk factors, suggesting that AIP may reflect not only metabolic imbalance but also the atherothrombotic environment that produces “hard” outcomes [36, 38]. There are even prospective data showing that cumulative exposure to high AIP over time is linked to higher long‐term risk of myocardial infarction, stroke, and composite cardiovascular events, implying that sustained atherogenic dyslipidemia plus low HDL‐C leaves a lasting vascular imprint that persists beyond a single laboratory value [38]. In parallel, higher AIP has been associated with hypertension and with diabetic microvascular complications such as diabetic kidney disease, reinforcing that AIP tracks both macrovascular and microvascular injury pathways in cardiometabolic disease [39, 40].

In our multi‐ethnic sample, the association between higher AIP and prevalent T2DM was most evident in the Fars subgroup, while estimates in several smaller ethnic groups were imprecise. The age‐ and sex‐adjusted AIP‐by‐ethnicity interaction suggested possible heterogeneity; however, these findings should be interpreted cautiously because several ethnic subgroups had limited sample sizes and sparse T2DM events. The observed pattern raises a hypothesis that genetic background, dietary patterns, or other sociocultural and environmental exposures may modify the relationship between lipid patterns and glycemic status. Future studies with adequate sample sizes in each ethnic group are needed to confirm or refute this possibility.

In additional exploratory subgroup analyses, the association between AIP and prevalent T2DM did not significantly differ by sex or age group. An exploratory interaction was observed by coronary lesion severity, with stronger associations in patients with 1‐vessel and 2‐vessel disease than in those with 3‐vessel disease. However, because these analyses were not the primary focus of the study and may be affected by subgroup size, residual confounding, and multiple testing, they should be interpreted cautiously. Exploratory ROC analysis also showed that AIP alone had limited discriminatory performance for prevalent T2DM; therefore, we did not propose a standalone clinical cutoff or recommend AIP as a screening tool.

This study has some limitations. First, HbA1c measurements were not consistently available, so we could not evaluate the association between AIP and longer‐term glycemic control. Second, some ethnic subgroups were relatively small, which limited the precision of ethnicity‐stratified estimates; therefore, these analyses should be interpreted as exploratory. Third, the cross‐sectional design precludes any inference about temporality, causality, or prediction. Fourth, although we additionally adjusted for lipid‐lowering medication use, residual confounding by medication dose, duration, adherence, and treatment indication may remain. Glucose‐lowering medication use was part of the T2DM definition and was therefore not included as a separate covariate in the primary T2DM models. Because all participants were undergoing coronary angiography, these findings may not generalize to asymptomatic community populations. Insulin levels and HOMA‐IR were not available; therefore, we could not directly assess whether insulin resistance mediated the association between AIP and prevalent T2DM. Finally, the subgroup and ROC analyses were exploratory; the subgroup findings require confirmation in independent samples, and the ROC analysis was not intended to derive a validated clinical cutoff.

## 5 Conclusions

In this cross‐sectional analysis of patients with PCAD, higher AIP was associated with prevalent T2DM and higher FBS after adjustment for demographic, lifestyle, cardiometabolic covariates, and lipid‐lowering medication use. Exploratory ethnicity‐stratified analyses suggested possible heterogeneity, with the association most evident in the Fars subgroup; however, estimates in several smaller ethnic groups were imprecise. These findings should be interpreted as hypothesis‐generating and do not establish causality or predictive utility. Prospective studies are needed to determine whether AIP adds value for future dysglycemia risk assessment in patients with PCAD.

## Author Contributions

**Reza Amani‐Beni:** formal analysis, software, methodology, writing – original draft. **Ghazal Ghasempour Dabaghi:** data curation, writing – original draft, software. **Noushin Mohammadifard:** project administration, methodology, supervision, conceptualization, writing – review and editing. **Ehsan Zarepur:** conceptualization, data curation, writing – review and editing. **Bahar Darouei:** methodology, writing – original draft. **Mehrdad Rabiee Rad:** formal analysis, writing – review and editing. **Fahimeh Haghighatdoost:** data curation, writing – review and editing. **Fatemeh Nouri:** resources, writing – review and editing. **Nizal Sarrafzadegan:** project administration, supervision, data curation, writing – review and editing. All authors have read and approved the final manuscript.

## Funding

This study was funded by the Research and Technology Department, Iran Ministry of Health and Medical Education, and the Iranian Network of Cardiovascular Research (grant number: #96110).

## Ethics Statement

The protocol was reviewed and approved by the Ethics Committee of Isfahan University of Medical Sciences (IR.MUI.REC.1396.2.055).

## Consent

All participants gave written informed consent before enrollment.

## Conflicts of Interest

The authors declare no conflicts of interest.

## Supporting information


**Table S1:** Exploratory subgroup analyses of the association between continuous AIP and prevalent T2DM.
**Figure S1:** Exploratory ROC curve of AIP alone for prevalent T2DM.

## Data Availability

The data that support the findings of this study are available from the corresponding author upon reasonable request.
