# Supplementary material for: The Association Between Atherogenic Index of Plasma and Type 2 Diabetes Mellitus in a Multi‐Ethnic Population of Premature Coronary Artery Disease Patients: Insights From IPAD Study
Source: Endocrinol Diabetes Metab. 2026 Aug 12;9(5):e70280. doi: 10.1002/edm2.70280 (PMC13463314; doi:10.1002/edm2.70280)
Supplement: Supplementary file 1 [file EDM2-9-e70280-s001.docx]

Supplementary Table S1. **Exploratory subgroup analyses of the association between continuous AIP and prevalent T2DM**

| Subgroup | n/T2DM cases | Crude OR per 1-unit AIP (95% CI) | P | Fully adjusted OR per 1-unit AIP (95% CI) | P | P for interaction |
| --- | --- | --- | --- | --- | --- | --- |
| **Female** | 572/282 | 4.06 (2.05, 8.03) | <0.001 | 2.60 (1.24, 5.44) | 0.011 | 0.161 |
| **Male** | 1047/305 | 2.28 (1.32, 3.92) | 0.003 | 1.64 (0.91, 2.95) | 0.098 |  |
| **Age <55 years** | 715/218 | 2.29 (1.21, 4.36) | 0.011 | 1.80 (0.88, 3.70) | 0.109 | 0.697 |
| **Age ≥55 years** | 904/369 | 3.04 (1.74, 5.28) | <0.001 | 2.23 (1.23, 4.04) | 0.008 |  |
| **1-vessel disease** | 718/219 | 4.67 (2.39, 9.13) | <0.001 | 4.92 (2.32, 10.43) | <0.001 | 0.0002 |
| **2-vessel disease** | 406/150 | 3.83 (1.61, 9.13) | 0.002 | 2.86 (1.12, 7.34) | 0.029 |  |
| **3-vessel disease** | 470/204 | 0.81 (0.39, 1.68) | 0.572 | 0.45 (0.19, 1.06) | 0.067 |  |

AIP, atherogenic index of plasma; T2DM, type 2 diabetes mellitus; OR, odds ratio; CI, confidence interval. AIP was modeled as a continuous variable. The n/T2DM cases column is based on the fully adjusted model sample. Fully adjusted models included demographic, lifestyle, cardiometabolic covariates, and lipid-lowering medication use, excluding the stratification variable where appropriate. P for interaction was obtained by including the interaction term between AIP and the subgroup variable in the fully adjusted model. These subgroup analyses were exploratory.

Supplementary Figure S1. **Exploratory ROC curve of AIP alone for prevalent T2DM**

The ROC curve shows the discriminatory performance of AIP alone for prevalent T2DM among patients with premature coronary artery disease. The AUC was 0.567 (95% CI: 0.539, 0.596), indicating limited discrimination.
